# Interleukin-22 facilitates the interferon-λ-mediated production of tripartite motif protein 25 to inhibit replication of duck viral hepatitis A virus type 1

**DOI:** 10.1186/s13567-023-01188-4

**Published:** 2023-06-30

**Authors:** Hao An, Yumei Liu, Ming Shu, Junhao Chen

**Affiliations:** School of Public Health, Weifang Medical University, Weifang, 261042 Shandong China

**Keywords:** TRIM25, IL-22, IFN-λ, phosphorylation, STAT3

## Abstract

The innate immune system provides a defense against invading pathogens by inducing various interferon (IFN)-stimulated genes (ISGs). We recently reported that tripartite motif protein 25 (TRIM25), an important ISG, was highly upregulated in duck embryo hepatocyte cells (DEFs) after infection with duck viral hepatitis A virus type 1 (DHAV-1). However, the mechanism of upregulation of TRIM25 remains unknown. Here we reported that interleukin-22 (IL-22), whose expression was highly facilitated in DEFs and various organs of 1-day-old ducklings after DHAV-1 infection, highly enhanced the IFN-λ-induced production of TRIM25. The treatment with IL-22 neutralizing antibody or the overexpression of IL-22 highly suppressed or facilitated TRIM25 expression, respectively. The phosphorylation of signal transducer and activator of transcription 3 (STAT3) was crucial for the process of IL-22 enhancing IFN-λ-induced TRIM25 production, which was suppressed by WP1066, a novel inhibitor of STAT3 phosphorylation. The overexpression of TRIM25 in DEFs resulted in a high production of IFNs and reduced DHAV-1 replication, whereas the attenuated expression of IFNs and facilitated replication of DHAV-1 were observed in the RNAi group, implying that TRIM25 defended the organism against DHAV-1 propagation by inducing the production of IFNs. In summary, we reported that IL-22 activated the phosphorylation of STAT3 to enhance the IFN-λ-mediated TRIM25 expression and provide a defense against DHAV-1 by inducing IFN production.

## Introduction

The innate immune system plays an essential role in the defense against invading viruses and subsequent activation of adaptive immunity [1]. Type I interferon (IFN-I) is an important effector of the IFN-I signaling pathway and elicits multifaceted effects in the host’s innate defense [2–4] as well as antiviral and immunomodulatory effects [5, 6]. In the past decade, an important paradigm shift has occurred in how we consider the compartmentalization of viral responses into systemic versus mucosal responders, which was driven in large part by the discovery of type III IFNs, or IFN-λ [7]. IFN-λ activates the corresponding signal transduction pathway, such as janus kinase-signal transducers and activators of the transcription (JAK-STAT), and induces the expression of IFN-stimulated genes (ISGs) by binding to an interferon receptor composed of interleukin (IL)-10 receptor β and IL-28 receptor α on the cell surface to exert the antiviral function [8]. To activate JAK-STAT, different cytokines specifically interact with their respective receptors and induce receptor dimerization, which subsequently activates the receptor-associated tyrosine kinases, also known as JAKs [9]. Subsequently, some STAT family members, such as STAT1, 3, 4, 5, and 6, interact with the receptor-kinase complex and activate themselves [9]. STAT1 and STAT2 form a heterodimer, which translocates to the nucleus and binds to IFN-stimulated response elements (ISREs) together with the interferon regulatory factor 9 [10–14]. In addition, some other heterodimers derived from different combinations of STAT1, 3, 4, 5, or 6 also enter into the nucleus and bind to ISREs to initiate transcription by binding to specific sites in the promoters of ISGs [15–19]. A number of ISGs, such as the double-stranded RNA-activated protein kinase, myxovirus resistance, virus inhibitory protein, ISG15, and tripartite motif protein 25 (TRIM25), significantly inhibit specific phases of the viral cycle, including viral entrance, replication, assembly, and budding [20, 21].

As a member of the TRIM family, TRIM25 is an E3 ubiquitin ligase and contains three conserved N-terminal domains: one or two B-Boxes (B1/B2) domain(s), a really interesting new gene domain, and a coiled-coil domain [17]. TRIM25 generally has an important function against invading viruses [20–25], however, it can also suppress the retinoic-acid-inducible gene-I (RIG-I) signaling pathway. TRIM25 can enhance the stability of the proinflammatory-inducible protein HLA-F adjacent transcription 10 (FAT10) to strengthen the inhibitory effect of FAT10 on RIG-I, resulting in low production of inflammatory factors [26]. It has been reported that the expression of TRIM25 in DHAV-1-infected duck embryo hepatocyte cells (DEFs) was upregulated [27]; however, its specific role during infection and mechanism of upregulation have not been elucidated yet.

In the classic IFN-λ-stimulation model, IFN-λ triggers the phosphorylation of STAT3 to induce more than 300 ISGs to restrict viral replication [28, 29]. It has been reported that some IFN-induced cytokines, such as IL-27, play a crucial role in regulating the production of ISGs by activating the phosphorylation of STAT3 [30]. IL-22, an important member of the IL-10 family, also exerts its biological functions by activating STAT3 [31–33]. It has been reported that STAT3 signaling is linked to all the known biological functions of IL-22, in particular to the upregulation of antibacterial factors and products that promote tissue repair [28, 34, 35]. However, it is still unclear whether the IL-22 cytokine newly identified in duck and sharing 59.9% similarity with human IL-22 [36] regulates ISGs production by mediating the phosphorylation of STAT3. In addition, although the transcription of various cytokines, such as IL-1β, IL-2, IL-4, IL-8, and IL-10, was shown to be highly upregulated to defend the organism against DHAV-(1/3) infection [37–40], the variation of duck IL-22 after DHAV infection is still unclear. Here, we reported that duck IL-22 was highly facilitated after DHAV-1 infection both in vitro and in vivo, especially in liver tissue. IL-22 activated the phosphorylation of STAT3 and significantly enhanced the IFN-λ-induced expression of TRIM25 to inhibit DHAV-1 propagation.

## Materials and methods

### Cells and viruses

DEFs cells and HEK 293T cells (ATCC, LGC Standards, Wesel, Germany) were cultured at 37 °C in Dulbecco’s modified Eagle medium (DMEM; Gibco, Carlsbad, CA, USA) supplemented with 10% fetal bovine serum (FBS, Gibco), penicillin at a concentration of 100 U/mL, and streptomycin sulfate at 100 µg/mL in a humidified atmosphere containing 5% CO_2_. The DHAV-1 virulent strain, WF1107, (GenBank accession nos. MW462237) was sampled from an outbreak of severe duck viral hepatitis (DVH) in WeiFang city, Shandong province, China.

### Plasmids and transfection

The mRNA sequences of TRIM25 (GenBank accession nos. KY974316.1), IFN-λ (GenBank accession nos. KJ206897), and IL-22 (GenBank accession nos. MT360382.1) were synthesized by Tsingke Biotechnology (Beijing, China), and were then inserted into the pcDNA™3.1/V5-His B vector to obtain the pcDNA-TRIM25, pcDNA-IFN-λ, and pcDNA-IL-22 recombinant plasmids.

For DNA transfection, cells contained in a 24-well plate were transfected in culture medium using NanoJuice (Novagen, WI, USA) according to the manufacturer’s instructions. In brief, 0.8 µL of NanoJuice or 2.4 µL of NanoJuice-transfection enhancer were separately diluted into 64 µL of opti-MEM and were then mixed together and incubated at room temperature (25 °C) for 5 min. Subsequently, 0.25 µg of recombinant plasmids was added into this mixture, which was then incubated for 15 min at room temperature. The mixture was then added into DEFs or HEK 293T cells.

For siRNA transfection, cells contained in a 24-well plate were transfected using the RFect transfection reagent (BAIDAI, China) according to the manufacturer’s instructions. In brief, 6 pmol siRNA or 2 µL of RFect reagent were individually diluted into 60 µL of opti-MEM and were incubated at room temperature for 5 min. Then, the siRNA and RFect diluents were mixed together and incubated at room temperature for 20 min, and subsequently the mixture was added to DEFs cells.

### Overexpression and RNAi

To upregulate the expression of TRIM25 and IL-22, DEFs were seeded into a 24-well plate to grow to 80% confluence and were transfected with pcDNA-TRIM25 or pcDNA-IL-22 recombinant plasmid. At 48 h post-transfection (hpt), cell lysates were collected to measure the overexpression effect of TRIM25 and IL-22. For the RNAi assay, the siRNA of TRIM25 (5′-GGAAGUAAGAAGAAUGAAAtt-3′) was transfected into DEFs as described above, and the silencing effect was measured at different hours after transfection.

### Western blot analysis

Total protein was extracted using the radio immunoprecipitation assay (RIPA) buffer (50 mM Tris-HCl at pH 7.5, 150 mM NaCl, 1% Ipegal, 0.5% sodium desoxycholate and protease inhibitors). In brief, DEFs or homogeneous tissue product were lysed in the buffer for 15 min on ice and the lysed product was centrifuged at 10 000 × *g* at 4 °C or 10 min; then, the supernatant was used to test protein expression. The concentration of the extracted protein was measured using the BCA protein assay kit (Solarbio, Beijing, China). The protein samples were then subjected to sodium dodecyl polyacrylamide gel electrophoresis on a 12% polyacrylamide gel, and the target proteins were electroblotted onto a polyvinylidene fluoride (PVDF) membrane (Thermo Fisher Scientific). Subsequently, the PVDF membrane was blocked with 5% non-fat milk in TBST (500 mL of NaCl, 0.05% Tween 20, 10 mM TRIS-HCl, pH 7.5) for 1 h, and was then incubated with the indicated antibody. After incubation, the PVDF membrane was then visualized using a mixture of hydrogen peroxide and 3,3′-diaminobenzidine tetrahydrochloride (Sigma-Aldrich, St. Louis, MO, USA), and visualized using an enhanced chemiluminescence system (Bio-Rad, Hercules, CA, USA). Images were captured by the BIO-RAD ChemiDoc^™^ Imaging System (Bio-Rad, Redmond, WA, USA). The band density was quantified using Image J Software (version 1.8.0, National Institutes of Health (NIH), Bethesda, MD, USA) with normalization to the β-actin signal.

### Antibodies

Horseradish peroxidase (HRP)-conjugated goat anti-mouse antibody (bs-0774R-HRP), HRP-conjugated goat anti-rabbit antibody (bs-40295G-HRP), rabbit anti-STAT3 antibody (bs-1141R), and rabbit anti-phospho-STAT3 (Ser727) antibody (bsm-52210R) were purchased from Bioss antibodies (Beijing, China). The monoclonal anti-β-actin antibody 8H10D10 was purchased from Cell Signaling Technology (Danvers, Massachusetts, USA).

The antisera against TRIM25, IL-22, and DHAV were produced in mice immunized with various purified proteins. The VP3 gene of DHAV-1, TRIM25, and IL-22 was inserted into the pET-32a(+) vector (Novagen, Darmstadt, Germany) to generate pET-VP3, pET-TRIM25, and pET-IL-22 recombinant plasmids, which was then separately transformed into *Escherichia coli* Rosetta (DE3) cells (Transgen Biotech, Beijing, China) to express VP3-His, TRIM25-His and IL-22-His fusion proteins. These fusion proteins were then purified as previously described and were used to produce polyclonal antibodies [41]. In brief, the 6-week-old Balb/c mice were separately primed subcutaneously with 100 mg of purified His-fusion proteins emulsified with an equal volume of Freund’s complete adjuvant (Sigma-Aldrich, St. Louis, MO, USA), and two booster immunizations with the fusion proteins in Freund’s incomplete adjuvant were administered at 2**-**week intervals. Finally, 100 mg of purified fusion proteins without adjuvant was used for immunization, and the polyclonal antisera were collected 72 h later.

### Purification of IFN-λ and IL-22

The HEK 293T cells transfected with IFN-λ-pcDNA or pcDNA-IL-22 were separately collected and sonicated on ice, and the insoluble fraction and cell debris were removed by centrifugation at 12 000 *g* for 1 min at 4 °C. The supernatant was then used to purify IFN-λ and IL-22 proteins by Ni^2+^ affinity chromatography using the His-Bind Resin chromatography kit (Novagen, Madison, WI, USA) according to the manufacturer’s instructions. In brief, the supernatant was purified on a gravity-flow column filled with 3 mL of Ni^2+^-NTA resin slurry (Novagen), and the His-tagged proteins were eluted in 50 mM NaH_2_PO_4_, 300 mM NaCl, and 300 mM imidazole (pH 8.0).

### qRT-PCR

For measurements related to DHAV-1, viral RNA was extracted using a E.Z.N.A.^™^ viral RNA kit (Omega Bio-Tek, Norcross, GA, USA) according to the manufacturer’s instructions. The extracted viral RNA was then quantified through a TaqMan real-time PCR assay as previously described [42]. For measurements related to cellular genes, total RNA was extracted using a total RNA kit (Omega Bio-Tek, Norcross, GA, USA) following the manufacturer’s instructions, and was then used for reverse transcription (TOYOBO, Japan). The cDNA obtained was subsequently used for qRT-PCR analysis via a 7500 Fast Real-Time PCR System (Applied Biosystems, Carlsbad, CA, USA) and SYBR Green PCR kit (TOYOBO). Amplification was performed in 20-µL reaction volumes according to the manufacturer’s instructions at the following cycling conditions: one cycle at 95 °C for 30 s (denaturation), 40 cycles at 95 °C for 5 s (annealing), and 34 s at 60 °C (extension), followed by a dissociation curve analysis step. GAPDH (F: ATGTTCzGTGATGGGTGTGAA; R: CTGTCTTCGTGTGTGGCTGT) was used to normalize the transcript levels. The primers used in the present research are listed in Table 1.

**Table 1:** **Real-time Q-PCR primers used for quantification of virus genomes and host cell gene**.

Target	Primer sequence (5′–3′)
IL-22	F: CAGGACACTGACAACAGGCT
	R: CCACCTCCTCAGTGTATGGG
TRIM25	F: GGAAAGCTAAGCCCCCTCAA
	R: TTGGTGCTCGGTTATCTGCC
IFN-α	F: TCCTCCAACACCTCTTCGAC R: GGGCTGTAGGTGTGGTTCTG
IFN-λ	F: ACCAGGCTCTTCAATCGGAA R: TTGGGTGAAGAAGGCCAGG
DHAV	F: AGACACATGTTGCTGAAAAACT R: TCCCCCTTATACTTAATGCCAG DHAV-1-Probe: Cy5-5′-ATGCCATGACACTAT CTCATATGAGTCAGC-3′-BHQ-2

### Experimental animals and sample collection

The 1-day-old cherry valley ducklings were purchased from a local commercial poultry farm (Weifang, Shandong, China). The 6-week-old Balb/c mice were purchased from a local Experimental Animal company (Jinan, Shandong, China). The treatment procedures of ducklings and mice were approved by the Experimental Animal Ethics Committee of Weifang Medical University (approval no. 2020SDL043) and performed in accordance with the guidelines of the Ethics Committee for Laboratory Animal Welfare of Weifang medical university. The experimental animals were provided a basal diet and water ad libitum and managed under the recommended humidity and temperature.

To measure the impacts of DHAV-1 propagation on expression level of IL-22 or TRIM25, the ducklings were divided into 2 groups, the experimental group was injected intramuscularly with 10^4.0^ copies of viral particles of DHAV-1, while the control group was injected intramuscularly with same volume of PBS. At 24 h post-infection (hpi), the ducklings were euthanized by carbon dioxide asphyxiation, 1 gram of liver, kidney, heart, spleen, thymus, and bursa of Fabricius were collected for total RNA extraction and qRT-PCR measurement. The immunized mice, which were used to produce antisera, were euthanized by carbon dioxide asphyxiation, followed by cervical dislocation. A total of 600 µL blood samples were collected from the eyeballs of each mouse, stored at room temperature for 2 h, and then were centrifuged at 3000 rpm/min to produce antisera.

### Statistical analyses

All experiments were performed at least three times with at least three biological replicates. Statistical significance was determined using SPSS software (version 20.0, SPSS Inc., Chicago, IL) evaluated using One-way ANOVA (3 or more groups of data) and independent-sample t test (only 2 group of data). *P < 0.05, **P < 0.01, ***P < 0.001.

## Results

### IL-22 expression was highly facilitated after DHAV-1 infection

**Figure 1:**
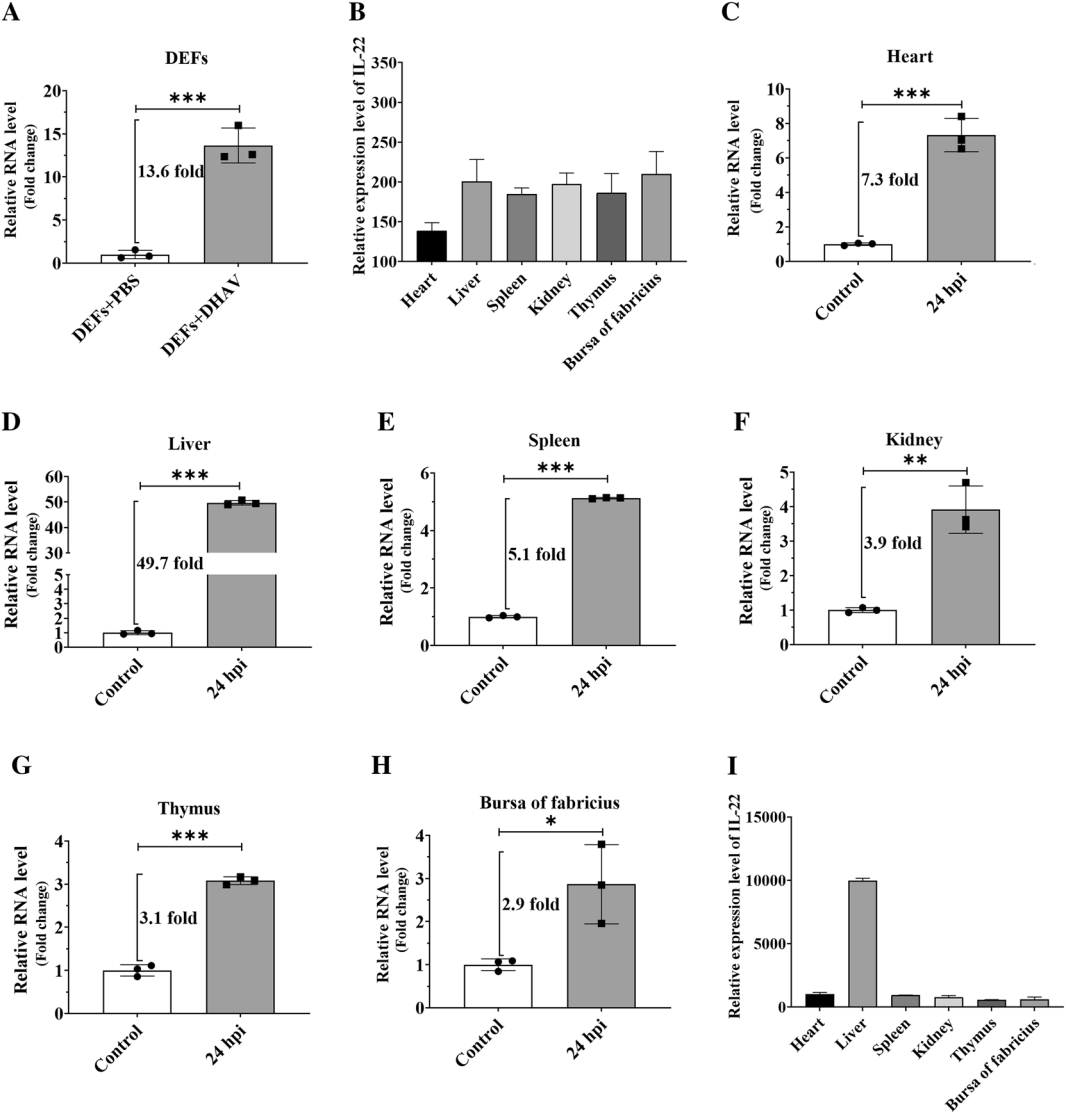
**The expression of IL-22 was highly facilitated in DEFs and various organs of ducklings.** **A** Experimental DEFs contained in a 6-well plate were inoculated with ~10^4.0^ copies of DHAV-1 particles, while those in the control group were treated with the same volume of PBS; the expression of IL-22 in both groups was then measured via qRT-PCR at 24 hpi. **B** IL-22 expression levels in heart, liver, spleen, kidney, thymus, and bursa of Fabricius of healthy 1-day-old ducklings obtained via qRT-PCR. **C–H** Ten healthy 1-day-old ducklings were divided into two groups, and ~10^4.0^ copies of DHAV-1 particles or same volume of PBS were injected intramuscularly; the IL-22 expression levels in the above-mentioned organs were measured via qRT-PCR at 24 hpi. **I** IL-22 expression levels in the above-mentioned organs measured at 24 hpi. The results are expressed as the mean ± SEM of three independent experiments. *P < 0.05, **P < 0.01, ***P < 0.001.

DHAV infection could lead to the different expression of various cytokines in young ducklings; for example, the transcription of IL-1β, IL-2, IL-4, IL-6, IL-8, and IL-10 was highly stimulated after infection [37–40]. However, it is still unknown how IL-22 varies at this stage and this variation was here measured in DEFs. Specifically, the experimental DEFs were inoculated with ~10^4.0^ copies of DHAV-1, while those in the control group were treated with the same amount of PBS, and the expressions of IL-22 in both groups were then compared at 24 hpi. The qRT-PCR results showed that IL-22 transcription in the DHAV-1-infected group was significantly higher (~13.6 times higher) than that in the control group (Figure 1A).

Then, the tissue distribution of IL-22 was measured in various organs of 1-day-old ducklings. IL-22 was detectable in all the tested organs: the bursa of Fabricius exhibited the highest expression level, followed by liver, kidney, spleen, thymus, and heart (Figure 1B). In addition, IL-22 was highly upregulated at all the tested tissues after DHAV-1 infection: the highest increments were observed in liver (~49.7-fold increase), and the lowest increments were observed in bursa of Fabricius (~2.9-fold increase) (Figures 1C–H). More importantly, the liver exhibited the highest expression level among all the tested organs after DHAV-1 infection (Figure 1I). These results showed that DHAV-1 infection significantly enhanced IL-22 production both in vitro and in vivo, especially in the liver.

### IL-22 directly inhibited DHAV-1 propagation by inducing the production of high amounts of IFN-λ

**Figure 2:**
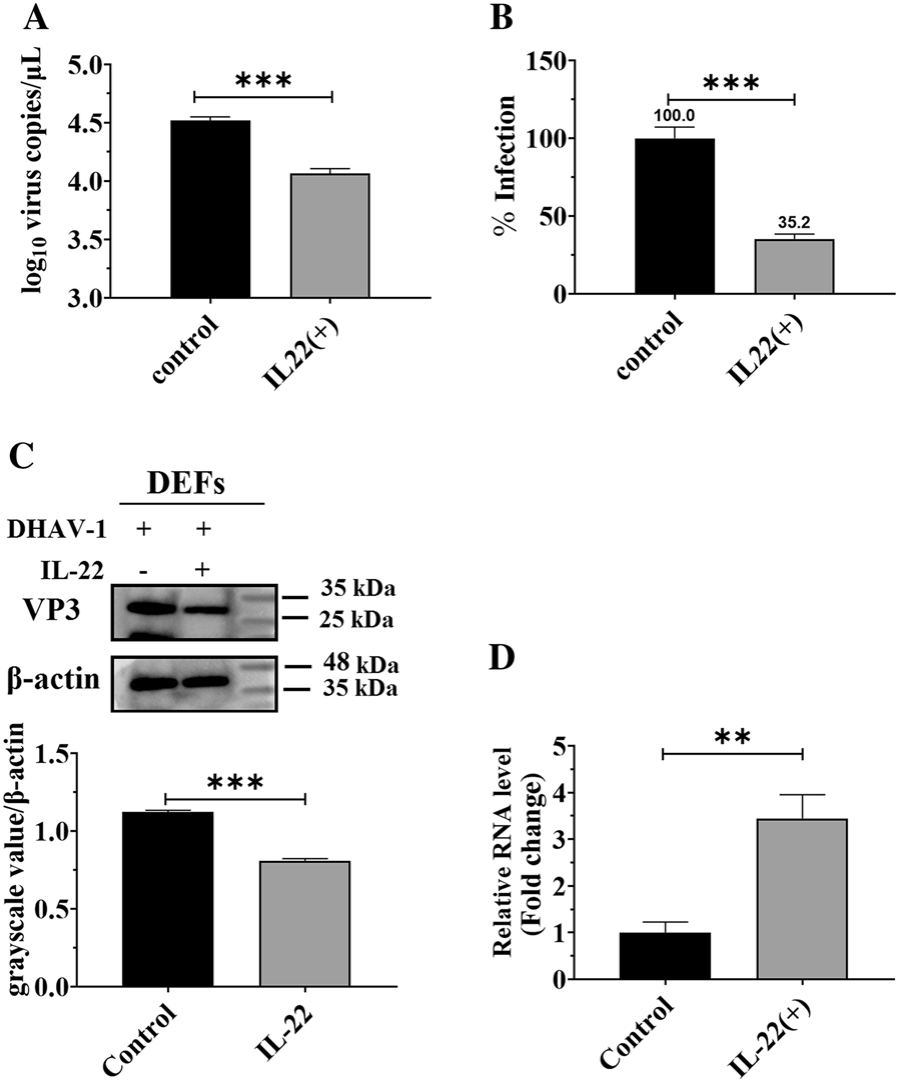
**IL-22 inhibited DHAV-1 replication by inducing the production of high amounts of IFN-λ.** **A** Experimental DEFs were treated with purified IL-22 at a concentration of 10 ng/mL and ~10^4.0^ copies of DHAV-1, while those in the control group were treated with the same amount of PBS and DHAV-1 particles; the viral replication levels in both groups were measured 24 h later via qRT-PCR. **B** The viral copies in the control group were set as 100%, the percentage of viral copies in experimental group was then indicated. **C** The DHAV-1 expression level in both groups was measured through Western blotting using anti-DHAV (1:100) and HRP-conjugated goat anti-mouse antibody (1:3000). The grayscale value of each band was measured using Image J software. **D** DEFs contained in a 6-well plate were inoculated with ~10^4.0^ copies of DHAV-1 or same amount of PBS; the expression of IFN-λ was measured via qRT-PCR. The results are expressed as the mean ± SEM of three independent experiments. **P < 0.01, ***P < 0.001.

IL-22 is an important member of the IL-10 family and plays a crucial role in mounting immune responses against various viral infections [29, 43, 44]. However, it is still unclear whether IL-22 provides protection against DHAV-1 infection in DEFs. Therefore, experimental DEFs were treated with purified IL-22 at a concentration of 10 ng/mL and ~10^4.0^ copies of DHAV-1, while those in the control group were treated with the same amount of PBS and DHAV-1 particles, and the viral replication levels in both groups were measured 24 h later. Compared with the control group, the treatment with IL-22 highly inhibited the propagation of DHAV-1 (Figure 2A) and reduced the infection by 64.8% (Figure 2B). In addition, the antiviral activity of IL-22 was also measured through Western blotting, and it was shown that viral protein expression was highly inhibited in the IL-22-treated group (Figure 2C).

Subsequently, the mechanism of defense against DHAV-1 provided by IL-22 was investigated. Previous reports have shown that IFN-λ and its receptor (IFN-λR) share homology with members of the IL-10 family and their receptors; in particular, the α-chain of the IL-22 receptor shares the closest relative with the IFN-λR1 chain (IFNLR1) [45, 46], which suggests that IL-22 might regulate IFN-λ expression. Here, our results showed that the treatment with IL-22 resulted in a significant increase in INF-λ production (Figure 2D). Overall, these data indicated that IL-22 inhibited the replication of DHAV-1 by inducing INF-λ expression.

### IL-22 enhanced the IFN-λ-induced production of TRIM25

**Figure 3:**
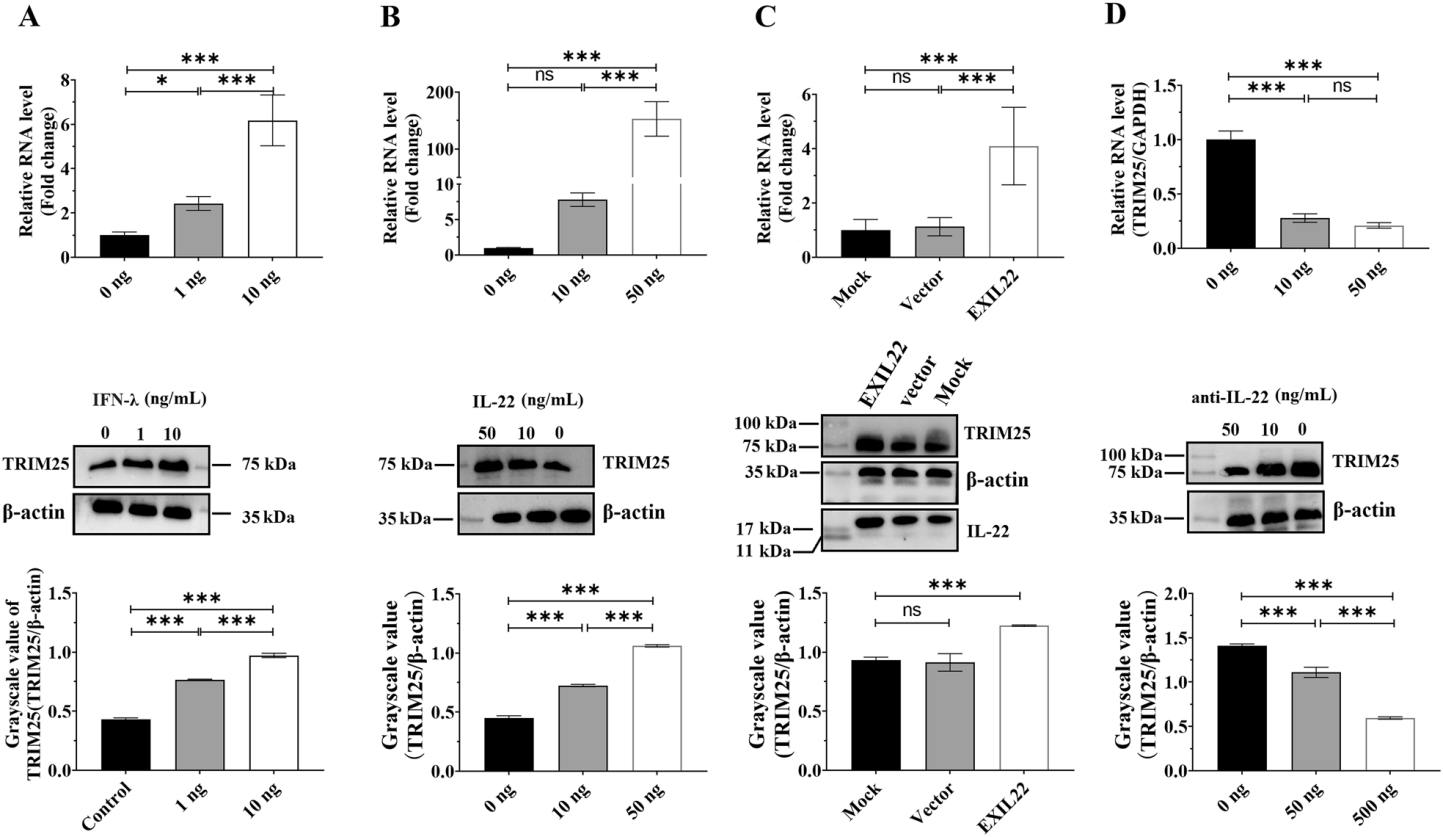
**IL-22 enhanced the IFN-λ-induced production of TRIM25.** **A** Treating DEFs with IFN-λ highly facilitated TRIM25 expression. DEFs were divided into three groups which were each treated with PBS, and purified IFN-λ at 1 ng/mL or 10 ng/mL for 24 h; TRIM25 expression was then measured via qRT-PCR and Western blotting using anti-TRIM25 (1:100) and HRP-conjugated goat anti-mouse antibody (1:3000). The grayscale value of each band was measured using Image J software. **B** The DEFs in the three groups were separately inoculated with PBS, purified IL-22 at 10 ng/mL or 50 ng/mL and purified IFN-λ at 10 ng/mL for 24 h, and the TRIM25 expression levels were measured via qRT-PCR and Western blotting. **C** The DEFs in the three groups were separately transfected with PBS, pcDNA3.1 vector, and pcDNA-IL-22, and the overexpression effect was measured at 48 hpt. Subsequently, purified IFN-λ at 10 ng/mL was added into the DEFs to induce TRIM25 expression, which was measured 24 h later via qRT-PCR and Western blotting. **D** The DEFs in the three groups were separately inoculated with PBS, IL-22 neutralizing antibody at 10 ng/mL and 50 ng/mL, and purified IFN-λ at 10 ng/mL for 24 h, and the TRIM25 expression levels were measured via qRT-PCR and Western blotting. The results are expressed as the mean ± SEM of three independent experiments. *P < 0.05, ***P < 0.001, ns represents *P* > 0.05.

INF-λ is an important component of the innate immune system, and viral infections activate both IFN-I and INF-λ through a common mechanism [8]. It has been reported that IFN-λ could participate in the antiviral defense by inducing the immediate transcription of ISGs, including TRIM25 [8, 28, 47, 48]. Therefore, we next investigated whether IFN-λ could induce TRIM25 expression in DEFs. DEFs cells were treated with different amounts of IFN-λ for 24 h, and the expression of TRIM25 was then measured via qRT-PCR and Western blotting. The results showed that TRIM25 was highly upregulated in a dose-dependent manner (Figure 3A), indicating that IFN-λ directly induced TRIM25 expression.

Previous research has reported that some IFN-induced cytokines could enter the cytoplasm to mediate TRIM25 expression [14, 30, 31, 33]. Therefore, it was here investigated whether IL-22 could mediate the IFN-λ-induced expression of TRIM25. Experimental DEFs were treated with different amounts of purified IL-22 and IFN-λ at a concentration of 10 ng/mL for 24 h, while those in the control group were treated with the same amount of PBS and IFN-λ, and the TRIM25 expression levels were then compared. The results showed that TRIM25 expression was highly facilitated compared to that in the control group (Figure 3B). In addition, the expression of IL-22 was upregulated through the transfection of pCDNA3.1-IL22 into DEFs cells, and IFN-λ was then added into DEFs at 24 hpt to induce expression of TRIM25. The same amount of pcDNA3.1 vector was used as negative control. Compared with the control group, the overexpression of IL-22 highly facilitated the production of TRIM25 (Figure 3C). Subsequently, experimental DEFs cells were treated with different amounts of IL-22-neutralizing antibody and IFN-λ at 10 ng/mL for 24 h, while those in the control group was treated with the same volume of PBS and IFN-λ, and the TRIM25 expression levels in both groups were then compared. The results showed that TRIM25 induction was inhibited in the antibody-treated group in a dose-dependent manner (Figure 3D). Collectively, these results indicated that IL-22 participated in TRIM25 induction.

### The phosphorylation of STAT3 was crucial for the IL-22-mediated production of TRIM25

**Figure 4:**
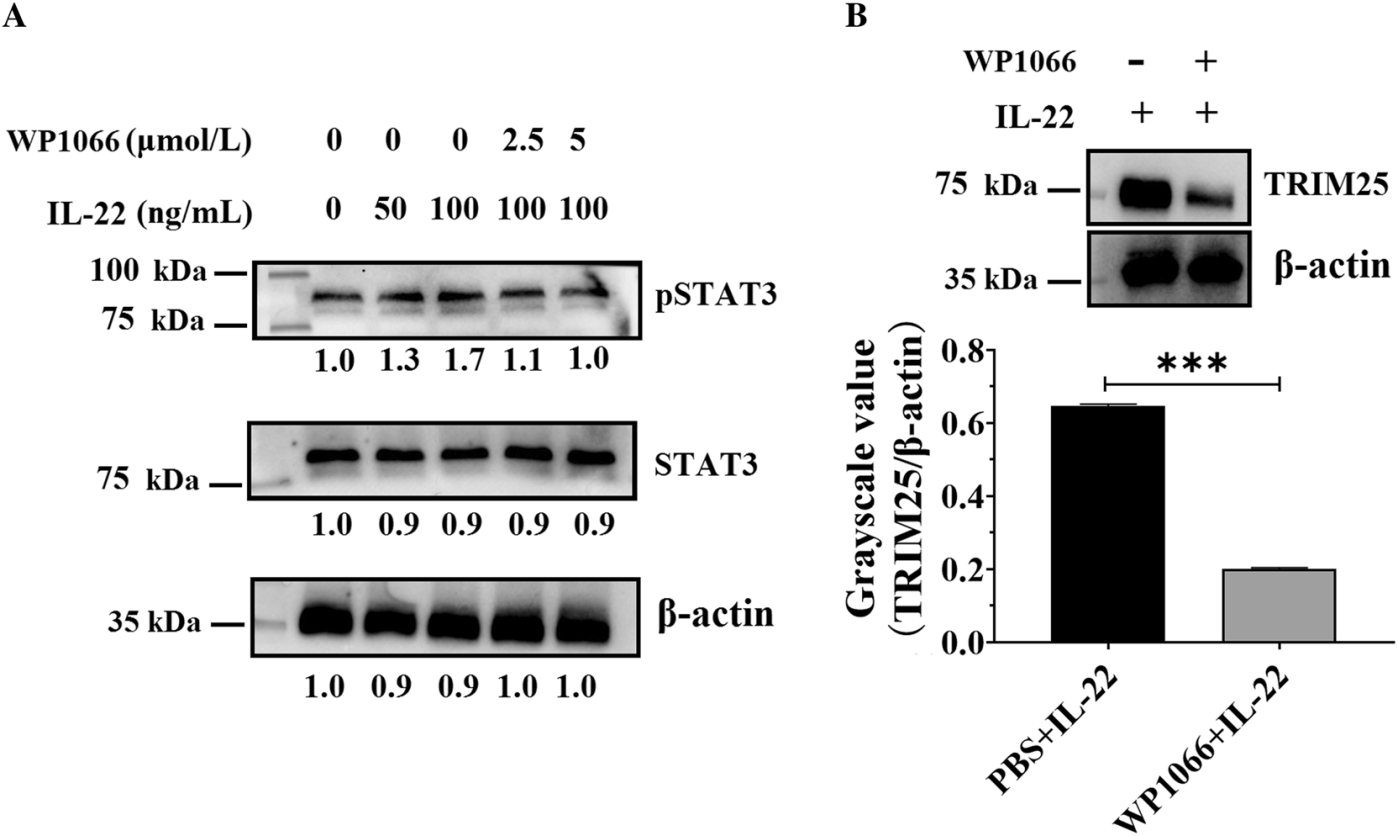
**The phosphorylation of STAT3 was crucial for the IL-22-mediated production of TRIM25.** **A** DEFs cells were stimulated with different amounts of IL-22 and WP1066 and were harvested 24 h post-treatment. Then, 30 mg of protein lysates were analyzed for P-STAT3 (S727) and total STAT3 via Western blot analysis (anti-pSTAT3 1:3000, anti-STAT3 1:3000, anti-β-actin 1:4000, HRP-conjugated goat anti-mouse antibody 1:3000). **B** The inhibition of P-STAT3 suppressed TRIM25 production. DEFs were treated with 5 µM WP1066 (or PBS in the control group) and IL-22 at 50 ng/mL for 24 h; TRIM25 expression was then measured via qRT-PCR and Western blotting. The results are expressed as the mean ± SEM of three independent experiments. *P < 0.05, ***P < 0.001.

Most of the biological functions of IL-22 are primarily mediated by the activation of the STAT3 signaling pathway downstream of IL-22R; IL-22 activates the phosphorylation of STAT3 and then triggers differential signal pathways [49]. Therefore, we next evaluated whether duck IL-22 enhanced TRIM25 production by mediating the phosphorylation of STAT3. We first investigated whether duck IL-22 could induce the phosphorylation of STAT3. DEFs were treated with different amounts of IL-22, and the phosphorylation level on Ser727 was determined 24 h later. The Western blotting results showed that IL-22 induced the phosphorylation of STAT3 in a dose-dependent manner (Figure 4A). In addition, DEFs were treated with different amounts of WP1066 (a novel inhibitor of STAT3 phosphorylation) and IL-22 at 50 ng/mL for 24 h, which caused the complete inhibition of phosphorylation (Figure 4A). These two results showed that IL-22 could activate the phosphorylation of STAT3.

Next, we investigated whether the phosphorylation of STAT3 was crucial for the IL-22-mediated production of TRIM25. Experimental DEFs were treated with 5 µM WP1066 and IL-22 at a concentration of 50 ng/mL for 24 h, while those in the control group were treated with equal amounts of PBS and IL-22, and TRIM25 expression was then measured in both groups. The results showed that it was highly inhibited in the WP1066-treated group compared with the control (Figure 4B), indicating that the phosphorylation of STAT3 was crucial for the IL-22-mediated production of TRIM25.

### TRIM25 hindered DHAV-1 replication

**Figure 5:**
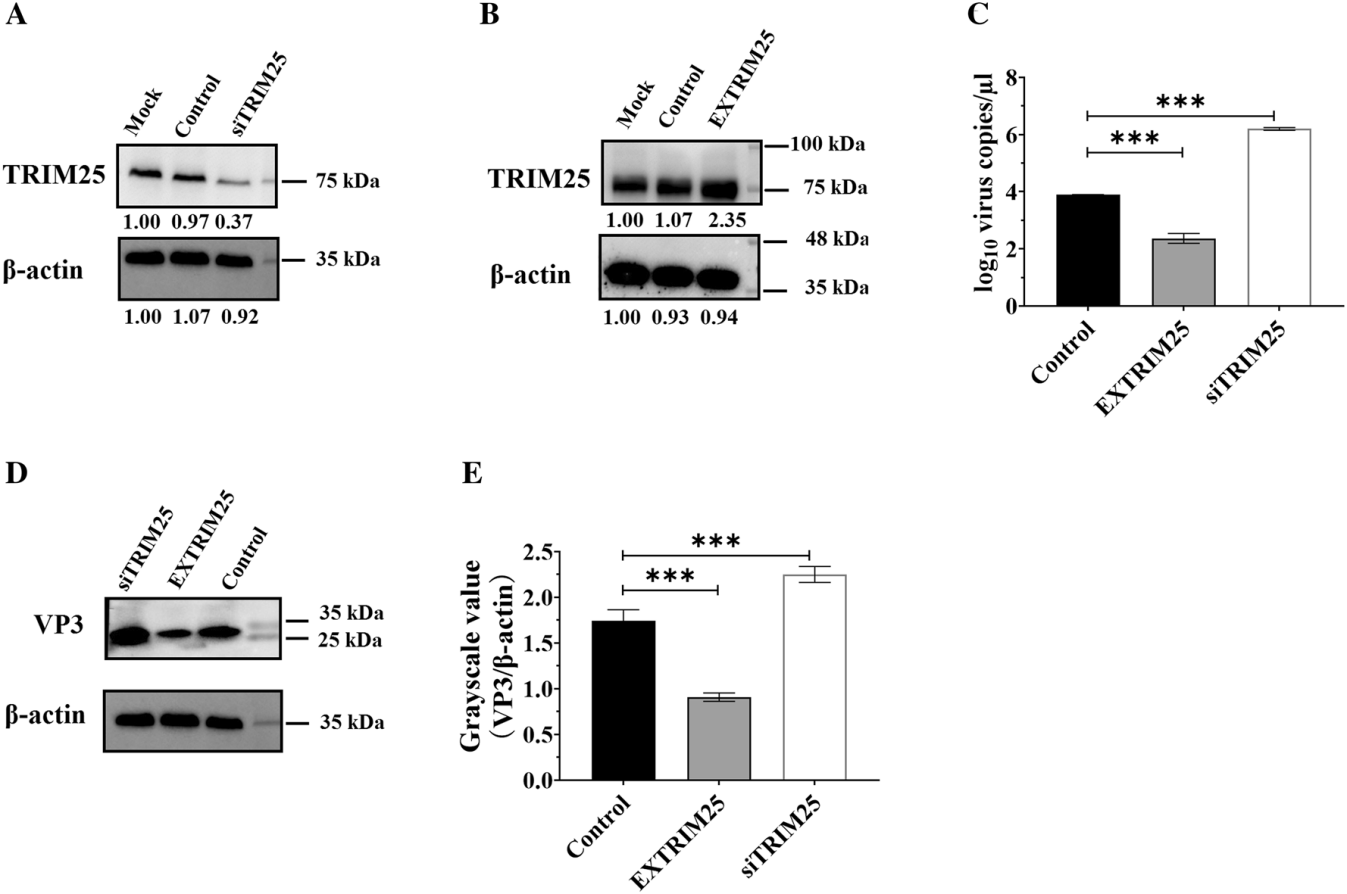
**TRIM25 highly inhibited DHAV-1 replication.** **A** TRIM25 was downregulated via RNAi assay and the silencing effect was measured at 72 hpt. **B** TRIM25 was upregulated by transfecting pcDNA-TRIM25 into DEFs. TRIM25 expression was highly facilitated at 48 hpt. **C** DEFs cells in the siTRIM25 group, EXTRIM25 group, and control group were then infected with ~10^4.0^ copies of DHAV-1 particles, and the viral growth curves for the different groups were obtained at 24 hpi via qRT-PCR. **D** TheDHAV-1 expression levels in the three groups were measured via Western blotting, anti-DHAV (1:100), and HRP-conjugated goat anti-mouse antibody (1:3000). **E** The grayscale values of DHAV-1 were measured. The results are expressed as the mean ± SEM of three independent experiments. ***P < 0.001.

To verify the functional role of TRIM25 during DHAV infection, its expression was upregulated (Figure 5A) via overexpression (named EXTRIM25 group) or downregulated (Figure 5B) via RNAi assay (named siTRIM25 group), respectively. DEFs cells in the siTRIM25 group, EXTRIM25 group, and control group were then infected with the same copies of DHAV-1 particles, and viral growth curves were obtained for the different groups at 24 hpi. Compared with the control group, RNAi against TRIM25 highly enhanced DHAV-1 replication, while the overexpression of TRIM25 significantly inhibited DHAV-1 propagation (Figures 5C–E), indicating that TRIM25 negatively regulated the viral replication.

### TRIM25 induced the expression of IFN-λ and IFN-I

**Figure 6:**
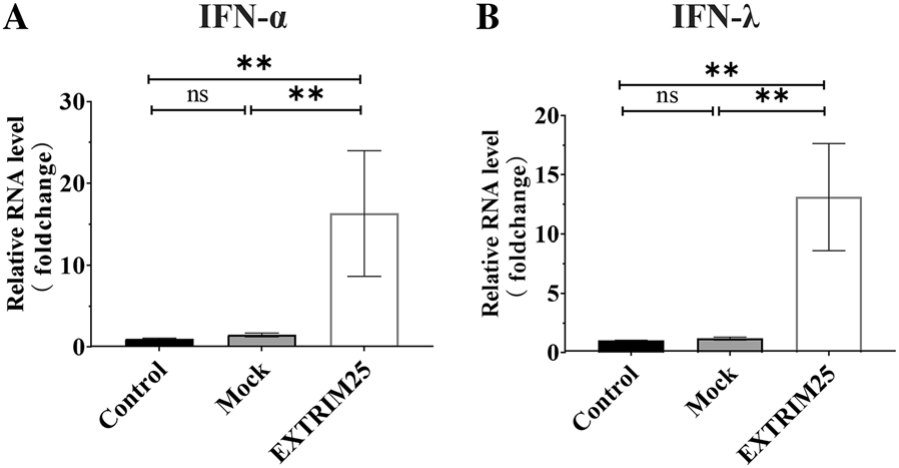
**TRIM25 enhanced the expression of IFN-λ and IFN-α.** The experimental group DEFs were transfected with recombinant plasmids pCDNA-TRIM25 to upregulate the expression level of TRIM25, while the control group were transfected with same copies of pCDNA3.1 vector, the expression levels of IFN-α and IFN-λ in both groups were measured via qRT-PCR. The results are expressed as the mean ± SEM of three independent experiments. **P < 0.01, “ns” represents *P* > 0.05.

Previous research indicated that TRIM25 is essential for the RIG-I-mediated production of IFNs and antiviral activity in response to infection with viral RNA [24]. However, whether TRIM25 inhibited DHAV replication by mediating IFN production is still unclear. The present study investigated the impact of TRIM25 on the production of INFs. TRIM25 expression was upregulated in DEFs, and the expression of INF-alpha (IFN-α) and IFN-λ was measured 24 h later. Compared with the control groups, the overexpression of TRIM25 resulted in a 16.3-fold increase in IFN-α expression (Figure 6A) and a 13.1-fold increase in IFN-λ production (Figure 6B).

### TRIM25 expression was highly facilitated after DHAV-1 infection

**Figure 7:**
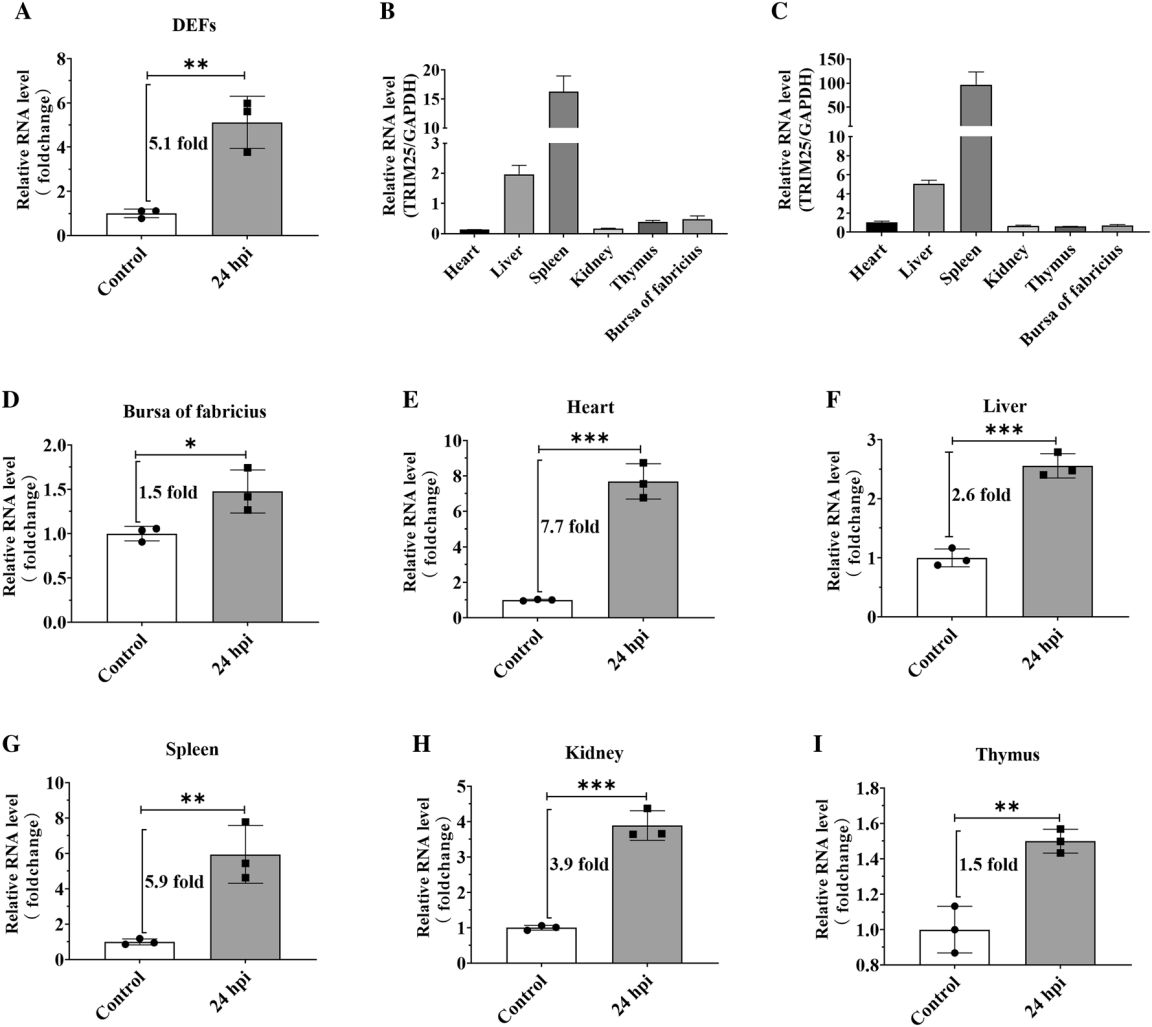
**TRIM25 expression was highly facilitated after DHAV-1 infection both in vitro and in vivo.** **A** DEFs were inoculated with ~10^4.0^ copies of DHAV-1 or PBS for 24 h, and TRIM25 expression was then measured via qRT-PCR. **B** Distribution of TRIM25 in the tissues of various organs of 1-day-old ducklings. **C** The expression of TRIM25 was measured in various organs of 1-day-old ducklings at 24 hpi. **D**–**I** Ten healthy 1-day-old ducklings were divided into two groups on average and ~10^4.0^ copies of DHAV-1 particles or same volume of PBS were injected intramuscularly; the TRIM25 expression levels in different organs were measured via qRT-PCR at 24 hpi.

TRIM25 is an important executor of the IFN signaling pathway and plays a crucial role in the defense against invading pathogens [50, 51]. Our previous results showed that TRIM25 expression was highly facilitated in DEFs after DHAV-1 infection [27], however, the specific increment had not been determined. In addition, the variation of TRIM25 in vivo after DHAV-1 infection is still unclear. Here, the impact of DHAV-1 propagation on TRIM25 expression in DEFs was first measured. DEFs were inoculated with ~10^4.0^ copies of DHAV-1, and TRIM25 expression was measured at 24 hpi. The qRT-PCR results showed a ~5.1-fold increase in TRIM25 expression compared with the control group (Figure 7A).

The impact of DHAV-1 replication on TRIM25 expression in 1-day-old ducklings was also investigated. The expression of TRIM25 was first measured in various organs of healthy 1-day-old ducklings, and the highest and lowest levels were observed in the spleen and heart, respectively (Figure 7B). Subsequently, the ducklings were injected intramuscularly with DHAV-1, and the expression of TRIM25 in various organs was determined (Figure 7C). Compared with the control group, TRIM25 expression was upregulated in all tested tissues after viral infection: the heart, liver, spleen, kidney, thymus, and bursa of Fabricius exhibited ~7.7, 2.6, 5.9, 3.9, 1.5, and 1.5-fold increases after DHAV-1 infection at 24 hpi, respectively (Figures 7D–I). Overall, TRIM25 expression was highly facilitated after infection both in vitro and in vivo.

## Discussion

DVH is an acute and fatal disease affecting young ducklings, mainly characterized by liver necrosis and hemorrhage that poses a significant threat to the domestic poultry industry. In recent years, outbreaks of DHV have been frequently reported in various countries, such as Egypt [52], Japan [53], and Vietnam [54]. Our previous results obtained from proteomic analysis showed that 507 proteins were differentially expressed after DHAV-1 infection (171 upregulated and 336 downregulated) [27]. TRIM25 was one of these proteins and was here chosen for further analysis. In addition, the functional role of IL-22, a newly identified cytokine, during DHAV infection was also investigated.

IL-22, a cytokine with structural homology to IL-10, is produced by active, adaptive, and innate components of the immune system, such as γδ T lymphocytes, macrophages, Th1, Th17, and Th22 cells [55, 56]. Recently, duck IL-22 was identified as an exocrine protein with a predicted molecular weight of 22.5 kDa, and was found to be highly expressed in the skin, followed by liver, heart, lungs, and brain [36]. IL-22 participates in preventing tissue damage, inducing tissue healing and regeneration [57]. For example, it inhibits the progression of viral-induced liver disease and hepatocellular carcinoma, and therefore reverses the injuries produced. Thus, it is considered as a useful tool to counteract the devastating effects of this tumor [57]. In addition, studies have shown that the proportion of natural lymphocytes highly increased to produce high amounts of IL-22, thereby promoting the phosphorylation of STAT3 signal in intestinal epithelial cells and playing a role in protecting the intestine from injury [58]. The results of the present study showed that the expression of IL-22 increased the most in liver tissue among all tested organs (Figure 1D); in addition, the highest expression of IL-22 after DHAV-1 infection was also observed in the liver (Figure 1I). Thus, it is hypothesized that IL-22 expression might be highly induced to prevent tissue damage and promote the healing of liver tissue after DHAV-1 infection. It has been reported that both STAT1 and STAT3 directly bind to the STAT responsive elements of the IL-22 promoter, and the balance between activated STAT3 and STAT1 determines the production of IL-22 [59]. A research on psoriasis pathogenesis revealed that STAT1 directly antagonized STAT3 and repressed IL-22 expression [52]. Here, we reported that IL-22 expression was highly facilitated after DHAV-1 infection both in vitro and in vivo (Figure 1), and the increase in expression might be due to duck STAT3 directly antagonizing STAT1 and leading to an increase in IL-22 promoter activities [59].

IL-22 plays a substantial role in the defense against invading pathogens, such as extracellular bacteria in the lung or gut [60–62], mucosal viral infections [28, 43], and intestine viral infections [44]. This protective function is exerted by inducing various cytokines and proinflammatory molecules, including IL-1, IL-6, IL-8, IL-11, G-CSF, GM-CSF, and LPS binding protein [63–66]. The present study showed that IL-22 inhibited the propagation of DHAV-1 by inducing the production of high amounts of IFN-λ (Figure 2). This inhibitory effect may be due to IL-22 enhancing the antiviral activity of IFN-λ by synergistically amplifying the IFN-λ-induced STAT1 signal pathway [28]. In addition, the accumulation of IFN-λ derived by IL-22 could also boost the antiviral activity of this interferon [44].

To exert its biological functions, IL-22 binds at the cell surface to the heterodimeric transmembrane receptor complex composed of IL-22R1 and IL-10R2 [49], and then activates the phosphorylation of STAT3 to trigger the JAK/STAT, ERK, JNK, and p38 MAP kinase pathways [33]. However, the functional role of IL-22 during DHAV-1 infection has not been fully elucidated yet. This study showed that IL-22 activated the phosphorylation of STAT3 and then enhanced the IFN-λ-mediated production of TRIM25 to inhibit virus proliferation (Figure 3). STAT3 generally plays a positive role in regulating the expression of ISGs by regulating the expression of IFN-inducible genes, such as CXCL11 [67]. However, the impact of STAT3 on ISGs can be bi-directional, and STAT3 can also highly inhibit the IFN-mediated antiviral response [68]. In this study, the pretreatment of DEFs with WP1066 suppressed the process of IL-22 enhancing IFN-λ-induced TRIM25 production, indicating that STAT3 was phosphorylated by IL-22 and then it positively regulated ISG expression.

During infection with the hepatitis B virus, IL-27 was found to be critical for the IFN-I-mediated production of TRIM25 [30]; specifically, the knockout of IL27R1 or treatment with the IL-27-neutralizing antibody significantly inhibited TRIM25 expression. In the present study, we showed that IL-22 was also crucial but not absolutely essential for TRIM25 production, and treatment with the IL-22-neutralizing antibody in IFN-λ-treated DEFs highly inhibited TRIM25 expression, albeit not completely (Figure 3). This might be due to the fact that some other IL-10 family members, such as IL-10 [69], IL-19 [70], IL-24 [71], and IL-26 [72], could also activate the phosphorylation of STAT3 and induce ISGs expression.

RIG-I is a viral RNA sensor and plays a crucial role in inducing IFN-mediated, host protective innate immunity against viral infection, and TRIM25 was also found to be essential during this process [25]. Specifically, RIG-I is inactive in uninfected cells and its activation depends on the E3 ubiquitin ligase activity of TRIM25 [24, 25, 73]. In response to various viral infections, the active RIG-I attaches to K63-polyubiquitin chains, which are synthesized by TRIM25, to promote the transcriptional upregulation of IFNs [24, 25, 73]. However, TRIM25 can also negatively regulate the activation of RIG-I. FAT10 is a ubiquitin-like protein that may elicit TRIM25’s repressive function mainly under inflammation conditions [26, 73]. FAT10 can interact with the second CARD motif of RIG-I and sequester the active form of RIG-I into insoluble precipitate, thereby inhibiting the RIG-I-mediated production of IFNs [26]. TRIM25 can strengthen the process by stabilizing the FAT10 protein [26]. The present study showed that TRIM25 expression was highly facilitated both in vitro and in vivo after the treatment of DHAV-1 (Figure 7). In addition, TRIM25 could highly suppresses DHAV-1 propagation by inducing a high expression of IFN-λ (Figure 6). These data suggested that TRIM25 was responsible for the defense against DHAV-1 by activating, and not repressing, the RIG-I/MAVS signaling pathway. The same was indicated by previously obtained results showing that the expression of RIG-I was highly facilitated in response to DHAV-1 infection [38]. TRIM25 provides a defense against invading pathogens through various pathways; for example, it can mediate the ubiquitination of the cytosolic pattern recognition receptor RIG-I to regulate the production of IFNs, other cytokines, and antiviral molecules [74, 75]. Here, our results indicated that TRIM25 directly induced the production of IFN-α and IFN-λ (Figure 6), which then activated corresponding signal pathways and induced various ISGs to hinder DHAV-1 replication. In addition, TRIM25 could also mediate the ubiquitination of the zinc-finger antiviral protein, a host factor that inhibits viral replication by binding to viral mRNAs and halting the translation and/or promoting the degradation of target mRNAs, to suppress viral replication [21]. Finally, TRIM25 could bind to the viral ribonucleoproteins and impede the movement of RNA into the polymerase complex to inhibit the elongation of viral RNA chains [76].

In conclusion, we showed that DHAV-1 propagation highly facilitated the production of IL-22, which activated the phosphorylation of STAT3 to enhance the IFN-λ-mediated production of TRIM25, which in turn provided a defense against DHAV-1 by inducing the production of high amounts of IFN-α and IFN-λ.

## Data Availability

The datasets analyzed in this study are available from the corresponding author upon reasonable request.

## Abbreviations

ISGs: interferon -stimulated genes
TRIM25: tripartite motif protein 25
DEFs: duck embryo hepatocyte cells
DHAV-1: duck viral hepatitis A virus type 1
IL-22: interleukin-22
STAT3: signal transducer and activator of transcription 3
JAK-STAT: janus kinase-signal transducers and activators of the transcription
ISREs: IFN-stimulated response elements
RIG-I: retinoic-acid-inducible gene-I
DMEM: Dulbecco’s modified Eagle medium
hpt: hours post-transfection
hpi: hours post-infection
RIPA: radio immunoprecipitation assay
PVDF: polyvinylidene fluoride
HRP: Horseradish peroxidase
FAT10: HLA-F adjacent transcription 10
