# Correction: Interleukin-22 facilitates the interferon-λ-mediated production of tripartite motif protein 25 to inhibit replication of duck viral hepatitis A virus type 1

**DOI:** 10.1186/s13567-024-01307-9

**Published:** 2024-04-09

**Authors:** Hao An, Yumei Liu, Ming Shu, Junhao Chen

**Affiliations:** School of Public Health, Weifang Medical University, Weifang, 261042 Shandong China

**Correction: Veterinary Research (2023) 54:53** 10.1186/s13567-023-01188-4

Following publication of the original article [1], the authors updated the Funding section.

The incorrect Funding is:

This research was funded by Natural Science Foundation of Shandong Province (Grant Number 201910240196), National Key R&D Program of China (2019YFA0904900) and Shandong Provincial Youth Innovation Team Development Plan of Colleges and Universities (No. 2019-6-156, Lu-Jiao).

The correct Funding is:

This research was funded by Natural Science Foundation of Shandong Province (Grant Number ZR2020QC013), National Key R&D Program of China (2019YFA0904900) and Shandong Provincial Youth Innovation Team Development Plan of Colleges and Universities (No. 2019-6-156, Lu-Jiao).
